# Effects of RNAi-based silencing of chitin synthase gene on moulting and fecundity in pea aphids (*Acyrthosiphon pisum*)

**DOI:** 10.1038/s41598-019-39837-4

**Published:** 2019-03-06

**Authors:** Chao Ye, Yi-Di Jiang, Xin An, Li Yang, Feng Shang, Jinzhi Niu, Jin-Jun Wang

**Affiliations:** 1 Key Laboratory of Entomology and Pest Control Engineering, College of Plant Protection, Southwest University, Chongqing, China; 2 Academy of Agricultural Sciences, Southwest University, Chongqing, China

## Abstract

The pea aphid, *Acyrthosiphon pisum*, is an important agricultural pest and an ideal model organism for various studies. Chitin synthase (CHS) catalyses chitin synthesis, a critical structural component of insect exoskeletons. Here, we identified a *CHS* gene from *A*. *pisum*, *ApisCHS*. The *ApisCHS* expression profiles showed that *ApisCHS* was expressed in various developmental stages and in all tested tissues of *A*. *pisum*, including the epidermis, embryo, gut and haemolymph. Notably, *ApisCHS* exhibited peak expression in the middle of each nymphal period and was extremely highly expressed in the epidermis and embryo. RNA interference (RNAi) showed that ~600 ng of dsRNA is an effective dose for gene silencing by injection for dsRNA delivery; moreover, 1200 ng·μL^−1^ dsRNA induced *CHS* gene silencing by a plant-mediated feeding approach. A 44.7% mortality rate and a 51.3% moulting rate were observed 72 h after injection of ds*ApisCHS* into fourth-instar nymphs, compared with the levels in the control (injected with ds*GFP*). Moreover, a longer period was required for nymph development and a 44.2% deformity rate among newborn nymphs was obtained upon ingestion of ds*ApisCHS*. These results suggest that *ApisCHS* plays a critical role in nymphal growth and embryonic development in pea aphids, and is a potential target for RNAi-based aphid pest control.

## Introduction

The exoskeleton of insects consists of cuticles and plays a variety of functional roles, including in protection, support, movement and acting as a shield against environmental stresses^1^. However, exoskeletons with a rigid structure have limitations regarding the growth and development of insects. To overcome this problem, insects periodically form a new cuticle to replace the old one, in which chitin plays a key role^1^. Chitin is the second most abundant natural polysaccharide after cellulose. It is mainly synthesised by arthropods, nematodes and fungi, and widely distributed in nature^2^. In insects, chitin is traditionally known as a central component of the epidermis, trachea and peritrophic matrix (PM)^3^. However, recently, some studies have reported that genes related to chitin are expressed in ovaries, eggs and eggshell of several insect species^4–6^.

Chitin synthase (CHS), as the principal enzyme in the last step of the chitin biosynthesis pathway, has been shown to catalyse the biosynthesis of chitin^7^. The first cloned cDNA of *CHS* in insects was from *Lucilia cuprina*^8^. To date, the cDNAs of *CHS*s have been cloned and characterised from more than 20 insect species^9^, including dipterans^10^, lepidopterans^11^, coleopterans^12^, orthopterans^13^ and hemipterans^14^. Generally, two distinct chitin synthase genes, *CHS1* and *CHS2*, have been identified in most insects. *CHS1* has been found to be specifically responsible for the synthesis of chitin in the epidermis, trachea, eggs and eggshell, whereas *CHS2* is exclusively expressed in the midgut epidermal cells, involved in hydrolysis of the PM of insects^12,15^. However, numerous studies have suggested that the PM is absent from phloem-sucking hemipterans, resulting in the disappearance of *CHS2* from their genome^12,16^. Furthermore, two alternative splicing variants of *CHS1* (*CHS1a* and *CHS1b*) were identified in *Locusta migratoria manilensis*^13^, which play distinct roles in different developmental stages. Subsequently, alternative splicing of *CHS1* was also identified in different insects, such as *Ostrinia furnacalis*^17^, *Bactrocera dorsalis*^18^, *Bombyx mori*^19^, *Nilaparvata lugens* and *Laodelphax striatellus*^20^. However, in aphids, no alternative splicing of *CHS1* occurs^9,21^.

Aphids, soft-bodied hemimetabolous insects, voraciously suck fluid from plants and are widely distributed globally. Approximately 250 aphid species are agricultural pests that cause enormous losses by sap-feeding and transmitting viral pathogens. Because of their small size, high fecundity and plasticity, aphids are considered to be the sucking pests that are the most difficult to control globally. The pea aphid, *Acyrthosiphon pisum*, as a model aphid, is a biological model for various types of study, and the completion of its whole genome sequence^22^ has provided an opportunity to develop new strategies with molecular tools for aphid control.

RNA interference (RNAi) is a powerful tool to study gene function in various organisms through the delivery of gene-specific double-stranded RNA (dsRNA); it is now also recognised as a next-generation approach for insect pest control^23,24^. In insects, both viral infections^25,26^ and nonspecific exogenous dsRNA^27,28^ can induce activity of the siRNA pathway. Because RNAi has been successfully used in many insect species including pea aphids^29,30^, it has been proposed as a potential strategy to help in solving problems in agricultural pest management^31,32^.

In this study, we performed molecular characterisation and expression profiling of *ApisCHS*, and carried out two dsRNA delivery methods for silencing this gene. We also reported the phenotypes in pea aphids upon the silencing of *ApisCHS*. The current study could deepen our understanding of the role of CHS in aphid growth as well as embryonic development, which should also guide further studies to develop this gene as a candidate for RNAi-based control of the pea aphid and other aphids.

## Results

### Sequence and phylogenetic analysis of *CHS*

**Figure 1:**
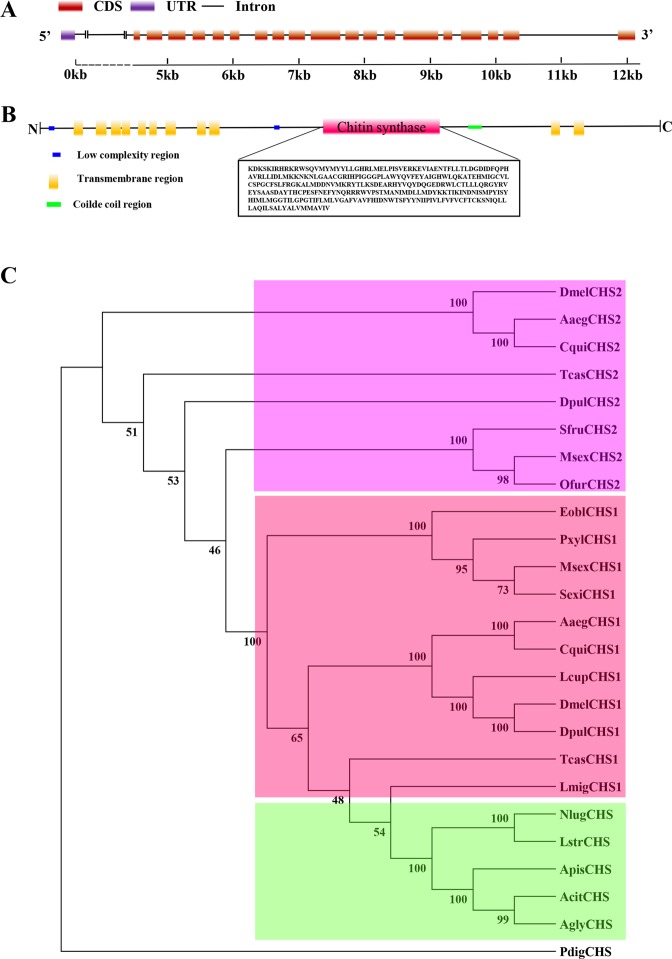
Sequence analysis of chitin synthase gene of *Acyrthosiphon pisum* and phylogeny of ApisCHS. (**A**) Schematic diagram of the genome sequence of *ApisCHS*. The CDS, UTR and intron are presented with different colours and patterns. (**B**) Confidently predicted domains are highlighted with different colours. A conserved sequence of 303 amino acids from ApisCHS is presented in the box using the online software SMART (http://smart.embl-heidelberg.de/). (**C**) Analysis based on the neighbour-joining method according to amino acid sequences using MEGA 6.0. Bootstrap support values with 1,000 samples are shown on the branches. Chitin synthases were from *Aedes aegypti* (Aaeg), *Acyrthosiphon pisum* (Apis), *Aphis glycines* (Agly), *Culex quinquefasciatus* (Cqui), *Daphnia pulex* (Dpul), *Drosophila melanogaster* (Dmel), *Ectropis oblique* (Eobl), *Laodelphax striatellus* (Lstr), *Locusta migratoria* (Lmig), *Lucilia cuprina* (Lcup), *Manduca sexta* (Msex), *Nilaparvata lugens* (Nlug), *Ostrinia furnacalis* (Ofur), *Penicillium digitatum* (Pdig), *Plutella xylostella* (Pxyl), *Spodoptera exigua* (Sexi), *Spodoptera frugiperda* (Sfru), *Aphis* (*Toxoptera*) *citricidus* (Acit) and *Tribolium castaneum* (Tcas) were chosen in this study. CHS of *Penicillium digitatum* was used as an outgroup.

The gene and protein structures of chitin synthase in *A*. *pisum* (*ApisCHS*) are displayed in Fig. 1A,B. With a full length of 4701 bp, *ApisCHS* has 18 exons and encodes a protein of 1566 amino acids. In terms of the protein structure, 11 transmembrane regions, 2 low-complexity regions and a coiled-coil region were identified. Based on the amino acid sequences of CHS from various insect species, a phylogenetic tree was constructed using MEGA6.0 to investigate the evolutionary relationship of *ApisCHS* among the selected insect species (Fig. 1C). In brief, CHS1 and CHS2 of various insect species are separated into two clusters. The CHSs of sap-sucking hemipterans all clustered with CHS1s, indicating that all of the presented CHSs belong to CHS1. To determine the relative expression of *ApisCHS* in various developmental stages and tissues of pea aphids as well as the expression of dsRNA targeting this gene, a qPCR detection region (266 bp) and RNAi target region (364 bp) were designed, which overlapped across a region of 86 bp, as shown in Fig. S2.

### Spatiotemporal expression profiles of *ApisCHS*

**Figure 2:**
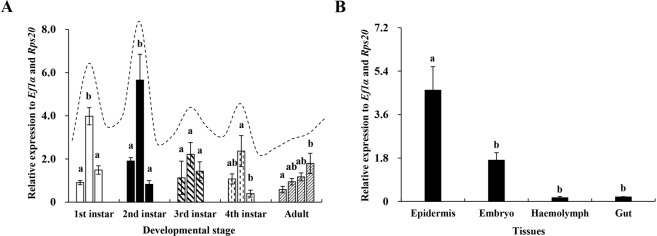
Spatiotemporal expression profiles of *ApisCHS* in pea aphids. (**A**) Relative expression of *ApisCHS* in different developmental stages. Substages (early, middle and late stages) were discriminated within each developmental stage. (**B**) Relative expression of *ApisCHS* in different tissues (including gut, embryo, epidermis, and haemolymph). In these experiments, we dissected the same tissues of 25 individual for each sample, with four biological replicates. The expression level (mean ± SE) was based on four biological replicates. Lower-case letters above each bar indicate significant differences among different treatments using one-way analysis of variance followed by Tukey’s test (*P* < 0.05).

The expression patterns of *ApisCHS* in different developmental stages and tissues were assayed by qPCR. The results showed that *ApisCHS* was generally expressed in all samples collected from various developmental stages (Fig. 2A). It is notable that *ApisCHS* was highly expressed in the samples collected at the middle of the first-instar as well as second-instar nymph stages. Notable expression of *ApisCHS* at the middle stage of third-instar as well as fourth-instar nymphs was also observed. Such expression peaks in the middle of each stage of the different instars may indicate the involvement of *ApisCHS* in development. In adulthood, the expression level of *ApisCHS* gradually increased over time. The expression level of *ApisCHS* was also analysed in various tissues; the results showed that it was mainly expressed in epidermis and embryo (Fig. 2B), with 2.91-, 24.12- and 29.15-fold higher levels in epidermis than in embryo, gut and haemolymph, respectively (*P* < 0.05). These results imply that *ApisCHS* is involved in nymphal and embryonic development and may mainly exert chitin-regulation-based functions in epidermis.

### RNAi-based silencing of *ApisCHS* via injection or ingestion of dsRNA

**Figure 3:**
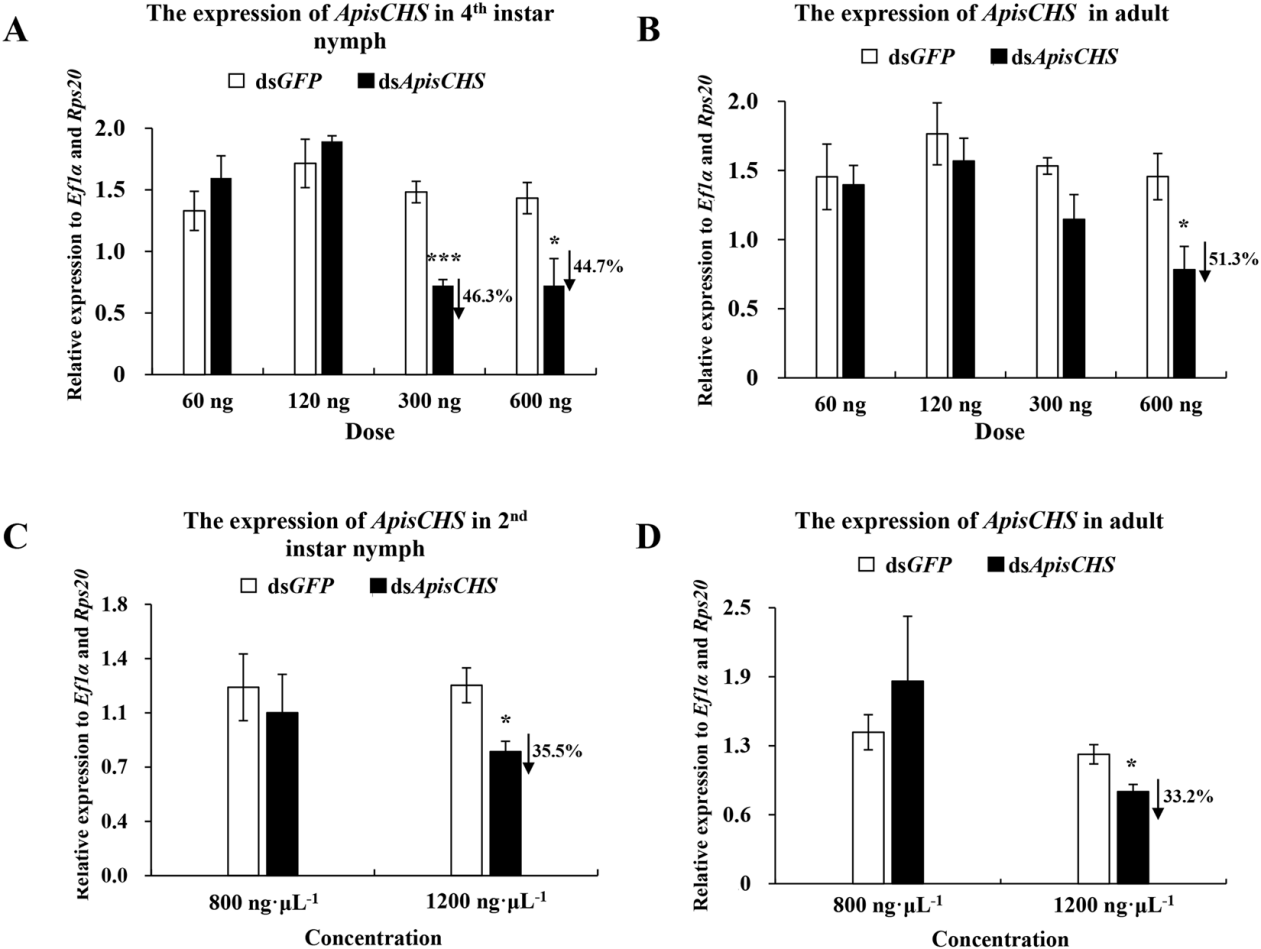
Silencing efficiency of *ApisCHS* upon different ds*ApisCHS* doses and concentrations via injection or ingestion. (**A**) Pea aphids were injected with 60, 120, 300 or 600 ng of dsRNA. RNA was extracted (four individuals included in each replicate) after 36 h. (**B**) Pea aphids ingested 800 and 1200 ng·μL^−1^ dsRNA. RNA was extracted (four individuals included in each replicate) after 36 h. Ds*GFP* was injected or ingested in the negative control. Statistical analysis was performed using Student’s *t-*test (mean ± SE; ^*^P < 0.05; ^***^P < 0.001).

To obtain an overview of the gene silencing achieved by the two dsRNA delivery methods (injection and ingestion) in pea aphids, different doses of ds*ApisCHS* were tested. Post 36 h dsRNA injection were used for detection of *CHS* expression were according to pre-experiment on time-points associated gene silencing efficiencies in pea aphids^33^. As shown in Fig. 3A, significant silencing of *ApisCHS* at 36 h after dsRNA injection (hpi) was achieved by using 300 or 600 ng of ds*ApisCHS* in fourth-instar nymphs. Both amounts resulted in about 46.3% and 44.7% reductions of the target gene, respectively. For adult aphids, significant silencing of *ApisCHS* at 36 hpi was detected only using 600 ng of ds*ApisCHS*. Specifically, knockdown of expression by 51.3% was confirmed (Fig. 3B). DsRNA ingestion was used for detection of *CHS* expression were according to pre-experiment in *A*. *citricidus*^9^. To test the feasibility of plant-mediated RNAi for aphids, two different concentrations of ds*ApisCHS* (800 and 1200 ng·μL^−1^) were applied to aphids at different developmental stages (from first-instar nymphs to adults). The silencing effects were detected in second-instar nymphs and adults; no silencing was observed unless the concentration of ds*ApisCHS* reached 1200 ng·μL^−1^ in both stages (Fig. 3C,D). At this concentration, significant reductions of *ApisCHS* of 35.5% and 33.2% were detected in second-instar nymphs and adults, respectively.

### Phenotype upon RNAi-based *ApisCHS* depletion through dsRNA injection

**Figure 4:**
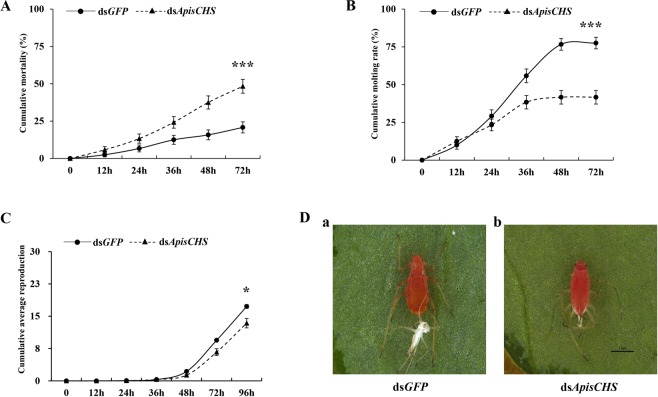
Effects of injection of ds*ApisCHS* on the development of *Acyrthosiphon pisum*. (**A**) Cumulative mortality after RNAi at 0, 12, 24, 36, 48 and 72 h. (**B**) Cumulative moulting rate in the treatment and control groups. Significance was analysed using the Kaplan–Meier method (mean ± SE; ^***^P < 0.001) in (**A**) and (**B**). (**C**) Cumulative reproduction after RNAi at the fourth-instar stage at different time points (0, 12, 24, 36, 48, 72 and 96 h). The mean (±SE) is based on three biological replicates. Statistical analysis was performed using Student’s *t-*test (^*^P < 0.05). (**D**) Phenotypes of *A*. *pisum* after the injection of ds*ApisCHS* for 48 h of (a) individuals alive and successfully moulted to adulthood and (b) individuals that died and failed to moult to adulthood.

To assess the effect of silencing *ApisCHS* on nymphal development, 300 ng of ds*ApisCHS* was injected into each nymph. The mortality and moulting were recorded for 72 h at 12-h intervals. We also determined the reproduction rate for each surviving adult. The results showed that cumulative mortality reached approximately 56.7% at 72 hpi, which was significantly higher than that in the control (25.8%, *P* < 0.001; Fig. 4A). Moreover, a moulting rate of approximately 41.6% was recorded in ds*ApisCHS*-injected aphids at 72 hpi, which was significantly lower than that in the control (77.5%, *P* < 0.001; Fig. 4B). Lethal phenotypes, such as treated nymphs becoming enwrapped in old cuticles and subsequently dying, were observed (Fig. 4D). The average reproductive output of ds*ApisCHS*-injected aphids was significantly lower than that in controls (*P* < 0.05; Fig. 4C) and abnormal newborn nymphs were identified, albeit at a low frequency, in ds*ApisCHS*-injected aphids (Fig. S2).

### Phenotype upon RNAi-based *ApisCHS* depletion through dsRNA ingestion

**Figure 5:**
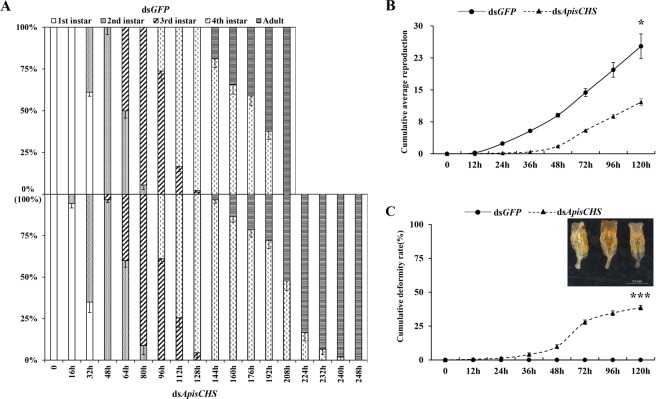
Effects of ingestion of ds*ApisCHS* on the development of *Acyrthosiphon pisum*. (**A**) The development time of *A*. *pisum* in the control and treatment groups. (**B**) The cumulative reproduction of adults in *A*. *pisum* based on three biological replicates. (**C**) Cumulative deformity rate of progeny after RNAi at different time points (0, 12, 24, 36, 48, 72, 96 and 120 h). Significance of differences analysed using the Kaplan–Meier method (mean ± SE; ^***^P < 0.001). The displayed phenotype of newborn nymphs after RNAi is shown in the upper right corner of the chart. Photographs were obtained using a Leica M205 C microscope (Leica Microsystems, Buffalo Grove, IL).

**Figure 6:**
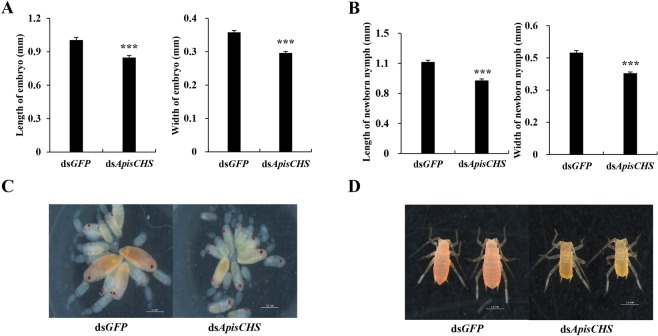
Phenotypes of embryonic development after silencing of *ApisCHS*. (**A**) Size of mature embryos (*n* = 60). Statistical analysis was performed using Student’s *t-*test (^***^P < 0.001). (**B**) Size of newborn nymphs (*n* = 60). Statistical analysis was performed using Student’s *t-*test (^***^P < 0.001). (**C**) Phenotypes of dissected embryos in treatment and control groups. (**D**) Phenotypes of dissected embryos in treatment and control groups. All photographs and sizes were obtained using a Leica M205 C microscope (Leica Microsystems, Buffalo Grove, IL) and measured by the software Leica Application Suite (LAS).

The results described above show the inhibitory effects of injection-based dsRNA delivery on aphid growth and reproduction through the silencing of *ApisCHS*. To further explore these effects of *ApisCHS* by a plant-mediated approach of ingesting dsRNA as a mimic of eating transgenic plants expressing ds*ApisCHS*, ds*ApisCHS* was administered orally in the stages from first-instar nymphs to adults. Under this set-up, the mortality observed in the above-mentioned injection-based ds*ApisCHS* treatment did not occur, but the development time and reproduction rate were altered. Specifically, the development time of nymphs in the treatment group was prolonged compared with that in controls (Fig. 5A). The average reproductive output was significantly decreased in the treatment group (*P* < 0.05; Fig. 5B). Meanwhile, the cumulative deformity rate of newborn nymphs was 44.2%, whereas the control group had a 0% deformity rate at 120 h (*P* < 0.001; Fig. 5C). Appendages of deformed newborn nymphs, include legs and antennas, could not unfold, resulting in death (Fig. 5C). Based on these results, we propose that the development of aphid embryos may be blocked upon the silencing of *ApisCHS*. To test this, we measured the body size of mature embryos and newborn nymphs (Fig. 6). We also recorded the numbers of embryos in the treatment and control groups (Fig. S4). The results showed that both newborn nymphs and embryos were significantly smaller than those in the control group (*P* < 0.001; Fig. 6). In addition, the number of embryos in the treatment group was reduced compared with that in the control group (*P* < 0.001; Fig. S4).

## Discussion

Chitin is an essential component of the exoskeletons of insects and plays key roles during growth and development. Chitin synthase is the core gene in the chitin synthesis pathway. Only one CHS gene was found to be present in aphids, which was named CHS1 (Fig. 1C). As a vital gene in the regulation of chitin synthesis in the aphids *A*. *glycines*^21^, *A*. *citricidus*^9^ and *Sitobion avenae*^34^, study of the function of this gene is helpful not only for understanding aphid development, but also for evaluating it as an RNAi target for potential use in pest control. However, in *A*. *pisum*, a model organism aphid, little study of CHS has been performed. Therefore, here, we first investigated the spatiotemporal expression of *ApisCHS* in pea aphids. In terms of temporal expression, *ApisCHS* was analysed in different developmental stages. Higher expression of *ApisCHS* was identified in mid-first- and mid-second-instar nymphs, and in mid-fourth-instar nymphs. We believe that chitin synthesis is required in these stages for the growth and moulting of aphids. Generally, epidermis was the main tissue where the expression of *CHS1* could be detected^17^; however, in recent research, the expression of *CHS1* was also detected in the eggs and ovaries of insects^13,14^. In the spatial analysis of expression, we confirmed that *ApisCHS* was expressed in epidermis and embryo. We thus hypothesised that *ApisCHS* may be involved in not only aphid development but also embryonic development. Therefore, we investigated the role of *ApisCHS* by RNAi via dsRNA delivery through injection as well as ingestion. In the delivery of dsRNA by injection, 46.3% silencing efficiency was detected in fourth-instar nymphs when a dose of dsRNA of 300 ng was delivered. For adults, the relative expression level of *CHS* was reduced by 51.3% when 600 ng of dsRNA was delivered. In addition, no downregulation of *CHS* was observed at the mRNA level in the fourth-instar nymph and adult stages when lower dsRNA doses (60 and 120 ng) were applied. These findings imply that a lower dsRNA dose could be the reason why no RNAi silencing was observed^35,36^. However, we encountered the problem that the microinjection was also harmful to fourth-instar nymphs and induced nonspecific mortality in pea aphids^37^. Compared with injection, ingestion is not only a more natural method to deliver dsRNA, but could also mimic transgenic plants for expressing ds*ApisCHS*, which enhanced the further step of applying RNAi as sucking-pest control^32,38^. The successful silencing of the target gene by administering dsRNA via ingestion was reported in pea aphids^39^. In the current study, we used *V. faba* seedlings as a vector to deliver dsRNA. Exogenous dsRNA was transported via the stem and leaves, and then ingested by aphids. The significant reduction of *ApisCHS* was detected when the dsRNA concentration reached 1,200 ng·μL^−1^; however, no gene silencing effect was detected when the dsRNA concentration reached 800 ng·μL^−1^. Although it is very difficult to accurately evaluate the amount of dsRNA ingested by aphids, it could be speculated that certain factors influence the efficiency of RNAi in considering the dsRNA delivery by plants. For instance, dsRNA in *V*. *faba* seedlings could be processed into siRNAs by plant RNAi machinery, which limited the efficacy of RNAi^40^; meanwhile, dsRNA could also be degraded by saliva of aphids^35^.

Generally, for homometabolous and hemimetabolous insects, the reduction of *CHS1* disrupts the growth of larvae or nymphs^12,20^. This result was also demonstrated by the phenotype associated with injection-based RNAi in the present study. *ApisCHS* was reduced at the fourth-instar nymph stage, leading to increased mortality and decreased moulting success. In contrast, such phenotypes were not observed in RNAi-based *ApisCHS* silencing in adults (Fig. S3). One possible explanation for this is that *CHS* plays a more critical role in the growth of nymphs than in adults, since adults are fully mature. However, another intriguing phenotype was identified: Some newborn nymphs born to the surviving fourth-instar nymphs that reached adulthood were deformed, although the deformity rate was very low. A similar phenotype was also observed by ingestion-based dsRNA delivery. A deformity rate in newborn nymphs of 44.2% was detected in aphids that ingested ds*ApisCHS*, together with reductions of the reproduction rate and the size of embryos as well as newborn nymphs. These results confirm that the development of embryos was blocked by ds*ApisCHS*. *CHS* is one of the most important factors required for embryonic development^41^. The disrupted synthesis of chitin by *CHS* through RNAi can disrupt embryonic development, leading to nymph malformation. Compared with the control ds*GFP* treated aphids, the ingestion of the dsRNA in targeting *ApisCHS* significantly delayed the overall development duration of the aphids. As we know, *ApisCHS* is highly expressed in the epidermis (Fig. 2B) and it is one of the most key genes to control the development of aphids^9,35^. By deleption of the expression of *ApisCHS* via ingestion of specific-dsRNA targeting *ApisCHS* from 1^st^ instar aphids until the adults, would inhibit the synthesis of chitin constantly and resulted in a cumulative effect in the overall development of the aphids, specially 3^rd^ instar to 4^th^ instar, and 4^th^ instar to adult (Fig. 5A). However, although extension of the developmental period of nymphs occurred, no mortality occurred from the stage of newborn nymphs to adults upon the ingestion of ds*ApisCHS*. A possible explanation for this is that the gene silencing efficiency of dsRNA-feeding-based RNAi is lower than for injection (Fig. 3), which was insufficient to induce severe phenotypes such as mortality.

In conclusion, in this study we investigated the involvement of *ApisCHS* in aphids and embryonic development through injection- or ingestion-based RNAi. We confirmed that *ApisCHS* plays a key role during the growth and embryonic development of pea aphids and silencing of this gene could lead to the inhibition of both aphid growth and embryonic development. In addition, although injection-based RNAi presented higher gene silencing efficiency than ingestion-based RNAi in our experimental set-up, injection is limited to only the study of gene function in the lab, while plant-mediated RNAi is not only a more natural method to deliver dsRNA, but could also be applied as a mimic of transgenic plants expressing ds*ApisCHS*, which would enhance the further step of applying RNAi as sucking-pest control. However, biosafety evaluation is indeed another important issue here. To further analyze this, the similarity of these targeted fragments in three aphid species, including *A*. *piusm*, *Myzus persicae*, and *Aphis glycines*, were checked by using DNAMAN (Lynnon Biosoft, CA, USA) (Fig. S5). These fragments showed an average of 95.70% similarity with each other. However, only ~68.17% similarities were obtained in comparison with beneficial insects, such as *Copidosoma floridanum* and *Apis mellifera*. The similarities among the aphids themselves and other beneficial insects showed a significant gap, which could be helpful to design an RNAi-based pesticide to target on various aphids while could be still safe to beneficial insects, but without strict biosafety evaluation procedure, it is still difficult to make such a conclusion in this case^42^. To date, plant-mediated RNAi has been applied in several aphid species, including *M*. *persicae*^43,44^, *S. avenae*^34,45^ and *A*. *citricidus*^9^. Especially in *S*. *avanae*^34^, the *CHS* was silenced via a plant-mediated RNAi and resulted in the unsuccessful moulting and reduced reproduction. These results are consistent with our study and also provide the potential method to use plant-mediated RNAi to manage aphid pest.

Here, successful plant-mediated RNAi for pea aphids demonstrated the potential of using RNAi for aphid control. Based on our research, further study should be performed to test the pest control capacity of constructing transgenic plants or engineering pathogenic fungi expressing aphid-specific dsRNA, such as CHS, to control aphids^45^.

## Materials and Methods

### Insects

*A*. *pisum* was collected as adults from an alfalfa field in Gansu Province, China, in 2016, and maintained in our laboratory. The stock colony was derived from a single parthenogenetic female, which was maintained on broad bean (*V*. *faba*) seedlings in a chamber at 22 ± 1 °C, ∼70% relative humidity, and a 16:8-h (light:dark) photoperiod. In our pre-experiment, we observed the entire developmental stages of the pea aphid in our lab condition. Based on this data, the 1^st^-, 2^nd^-, 3^rd^-, and 4^th^-instar nymph was divided into three substages, including early, middle and late stages, and four substages for an adult (newly emerged, early production, middle production, and late production stages) (Fig. S6). To clarify, the schematic diagram of the sample collection time points at each stage of development were shown in Fig. S6.

### Insect dissection and RNA extraction

Haemolymph, gut, epidermis and embryo were dissected from parthenogenetic adults in cold 0.01 M PBS (phosphate-buffered saline). Haemolymph was isolated by differential centrifugation, in accordance with the protocol of isolating haemocytes from *Drosophila melanogaster*^46^. All samples were kept in an ultra-low-temperature freezer at −80 °C. Total RNA of samples from the RNAi experiment was extracted using TRIzol^®^ reagent (Invitrogen, Carlsbad, CA, USA), except that RNA of haemocytes was extracted using TRIzol^®^ LS reagent (Invitrogen, Carlsbad, CA, USA). The integrity of RNA samples was evaluated on 1% (*w*/*v*) agarose gel by electrophoresis and quantified using a NanoDrop™ One microvolume UV-Vis spectrophotometer (Thermo Scientific, Wilmington, DE, USA).

### cDNA synthesis and cloning

Genomic DNA contaminating the samples was removed by DNase I (Promega, Madison, WI, USA). First-strand cDNA was synthesised using a PrimerScript®RT Reagent Kit (Takara, Dalian, China), following the manufacturer’s instructions. The cDNAs were stored at −20 °C until use. The specific primers (10 nM; Table S1) of *ApisCHS* were designed based on *A*. *pisum* genomic data, and the fragments of target and *GFP* genes were amplified with cDNA prepared from whole-aphid-body RNA isolates by PCR. The PCR conditions were as follows: initial denaturation at 95 °C for 3 min, followed by 38 cycles of 30 s at 95 °C, 30 s at 60 °C, and 45 s at 72 °C, and final extension of 7 min at 72 °C. The PCR fragments were gel-purified with a Gel Extraction Mini Kit (Takara) and ligated into pGEM-T easy vector (Promega). Recombinant plasmids were subsequently sequenced by an ABI Model 3100 automated sequencer (Invitrogen Life Technologies, Shanghai, China). Subsequently, the plasmids were cloned in LB broth medium for 24 h in a thermostatic oscillator at 37 °C and kept at 4 °C in a refrigerator before the subsequent synthesis of dsRNA.

### Synthesis and delivery of dsRNA

The dsRNA was synthesised using the TranscriptAid T7 High Yield Transcription Kit (Thermo Scientific), in accordance with the manufacturer’s instructions. The integrity of dsRNA was evaluated on 1% (*w*/*v*) agarose gel by electrophoresis and concentration using a NanoDrop™ One microvolume UV-Vis spectrophotometer (Thermo Scientific, Wilmington, DE, USA). The injection and ingestion of dsRNA were the two delivery methods used in the RNAi assays. The microinjection method to silence gene expression was performed using a M3301 micromanipulator (WPI Inc., Sarasota, FL, USA). Heated agarose (Invitrogen) was poured into a Petri dish and left until cooled, and then a knife was used to make two cross-shaped grooves for immobilising aphids. Glass capillaries (3.5-inch 3-000-203-G/X micropipettes; Drummond Scientific, Broomall, PA, USA) were prepared by a P-97 Micropipette Puller (Sutter Instrument Co., Novato, CA, USA) under the following parameters: Heat = 577, Pull = 145, VEL = 145, and time = 250. Injected aphids were placed on glass culture dishes (diameter = 250 mm) containing *V*. *faba* stem and inserted into a 1.5-mL centrifuge tube that was filled with water. For dsRNA delivery by ingestion, the concentrations of dsRNA were chosen based our previous study^9^ and *V*. *faba* seedlings were used to deliver dsRNA to inhibit the expression of the target gene based on a slightly modified version of the method reported for *Aphis* (*Toxoptera*) *citricidus*^9^. In brief, a 10–12-cm-long *V*. *faba* tender seedling was cut and inserted into a 1.5-mL centrifuge tube containing 1,000 μL of dsRNA (concentration 1,200 ng·μL^−1^) as a unit. Then, the unit was transferred into transparent plastic cups with small holes.

### Quantitative real-time PCR (RT-qPCR)

To monitor the relative expression level of the target genes, four biological replications were included in each treatment, as well as a control. Primers used for RT-qPCR (Table S1) analysis were designed by an online primer designing tool (available at https://www.ncbi.nlm.nih.gov/tools/primer-blast/). Before qPCR analysis, the specificity of the primers for RT-qPCR was investigated using a melting curve and a standard curve. RT-qPCR was performed in a 96-well plate with the CFX Connect™ Real-Time System and analysed by Bio-Rad CFX Manager software (both from Bio-Rad, Singapore). The qPCR was performed with a 10-μL reaction mixture containing 5.0 μL of NovoStart SYBR qPCR SuperMix (Novoprotein, Shanghai, China), 0.5 μL of forward and reverse specific primers, and 3.5 μL of nuclease-free water. PCR amplifications were performed with the following cycling conditions: 95 °C for 2 min, followed by 39 cycles of 95 °C for 15 s and 60 °C for 30 s. At the end of the PCR, a melting curve analysis was performed from 60 °C to 95°C with increases in 0.5°C increments, with 5 s of holding for each read. Two reference genes, *Rps20* and *Ef1α*, were used to normalize the expression levels of targeted genes through qBase+^47^ based on the 2^−ΔΔCT^ method. In addition, *Rps20* and *Ef1α* were examined the distribution of the Cq value in different treatments (Fig. S7A) and determined the stability of reference genes by BestKeeper (Fig. S7B)^48^.

### Statistical analyses

The data are expressed as the mean ± SE. Statistical analysis was performed using Student’s *t-*test or one-way analysis of variance, and the Kaplan–Meier test was used to test the significance of differences in mortality, reproduction and developmental duration using Statistical Package for the Social Sciences (SPSS) 22.0 software (IBM, Armonk, NY, USA).

### Ethics Statement

The research project was conducted on invertebrate species that are not subjected to any specific ethical issue and legislation.

## Supplementary information

Supplementary Information
