# Supplementary material for: Effects of RNAi-based silencing of chitin synthase gene on moulting and fecundity in pea aphids (Acyrthosiphon pisum)
Source: Sci Rep. 2019 Mar 6;9:3694. doi: 10.1038/s41598-019-39837-4 (PMC6403427; doi:10.1038/s41598-019-39837-4)
Supplement: Supplementary file 1 — Supplementary Information [file 41598_2019_39837_MOESM1_ESM.pdf]

Effects of RNAi-based silencing of chitin synthase gene on moulting and fecundity in pea aphids (*Acyrthosiphon pisum*)

Chao Ye[1, 2], Yi-Di Jiang[1, 2], Xin An[1, 2], Li Yang[1, 2], Feng Shang[1, 2], Jin-Zhi Niu[1, 2], and Jin-Jun Wang[1, 2, *]

[1] Key Laboratory of Entomology and Pest Control Engineering, College of Plant Protection, Southwest University, Chongqing, China;

[2] Academy of Agricultural Sciences, Southwest University, Chongqing, China.

[*] Correspondence: Jin-Jun Wang, Key Laboratory of Entomology and Pest Control Engineering, College of Plant Protection, Southwest University, Chongqing 400715, China. Tel.: +86-23-68250255; fax: +86-23-68251269; Email: wangjinjun@swu.edu.cn

Table S1. Primers used for dsRNA synthesis and RT-qPCR

Fragment	Forward (5’ to 3’)	Reversed (5’ to 3’)	PCR Type and efficiency
ds*ApisCHS*	[a] CATTGTCACGTGGGGTACGA	[a] CTGATGTGGGACGCTACTGG	[b] RT-PCR
ds*GFP*	[a] TGAGCAAGGGCGAGGAGCTG	[a] TCGATGCGGTTCACCAG	[b] RT-PCR
q*ApisCHS*	ACTGGGCGAGGACGGTATC	ACTCTTCGGCGGCTTTCTT	RT-qPCR [c] E=104.0
q*Ef1α*	CTGTGCTTATTGTCGCTGCT	TCGCTGTATGGTGGTTCAGT	RT-qPCR [c] E=99.7
q*Rps20*	AAGTGTGTGCTCCGAGATGA	CAGCAATGACACCGGGTTC	RT-qPCR [c] E=98.6

[a] The gene-specific parts of the primer are listed. These are preceded by the T7 adaptor TAATACGACTCACTATAGGG for dsRNA synthesis.

[b] These primers were used in RT-PCR for dsRNA synthesis.

[c] These primers were used in RT-qPCR for mRNA level detection. E: primers’ amplification efficiency (%).

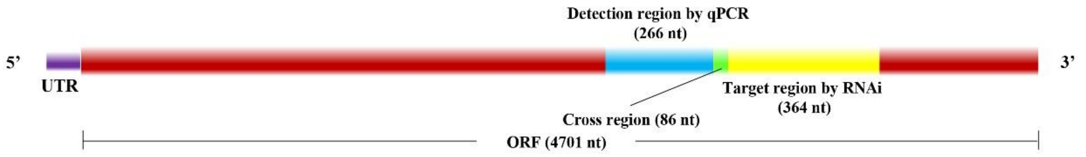

Figure S1. Schematic diagram of mRNA for RNAi of *ApisCHS*

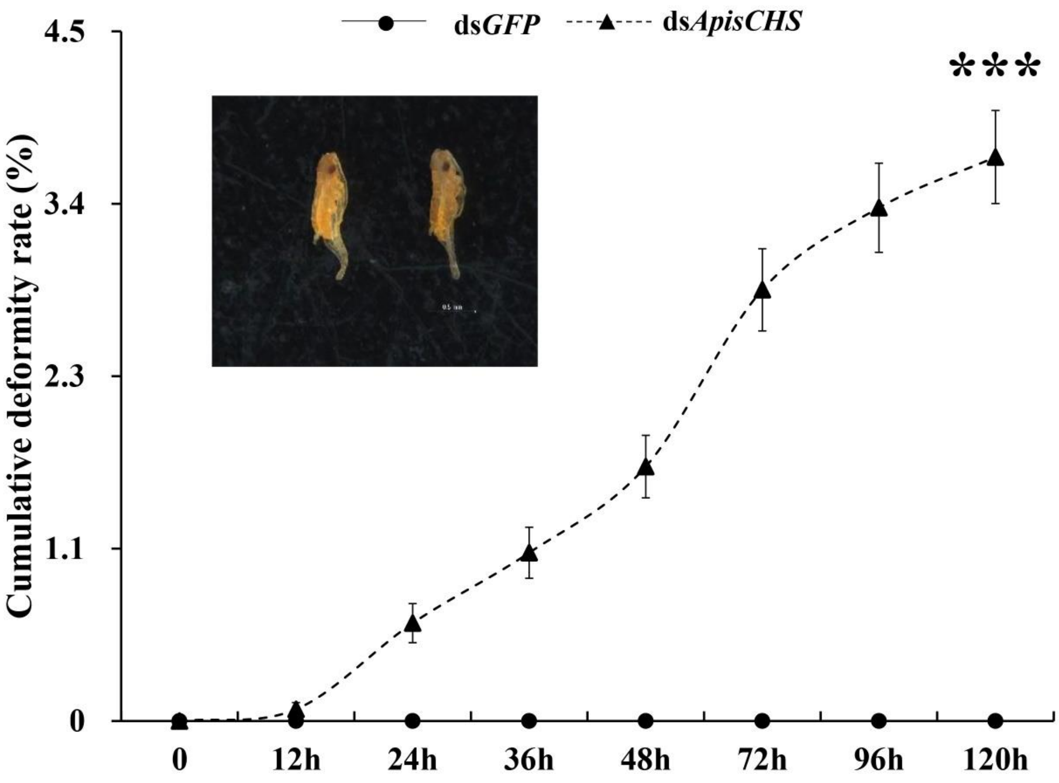

Figure S2. Cumulative deformity rate of progeny from adults which transition from 4th instar nymph by RNAi

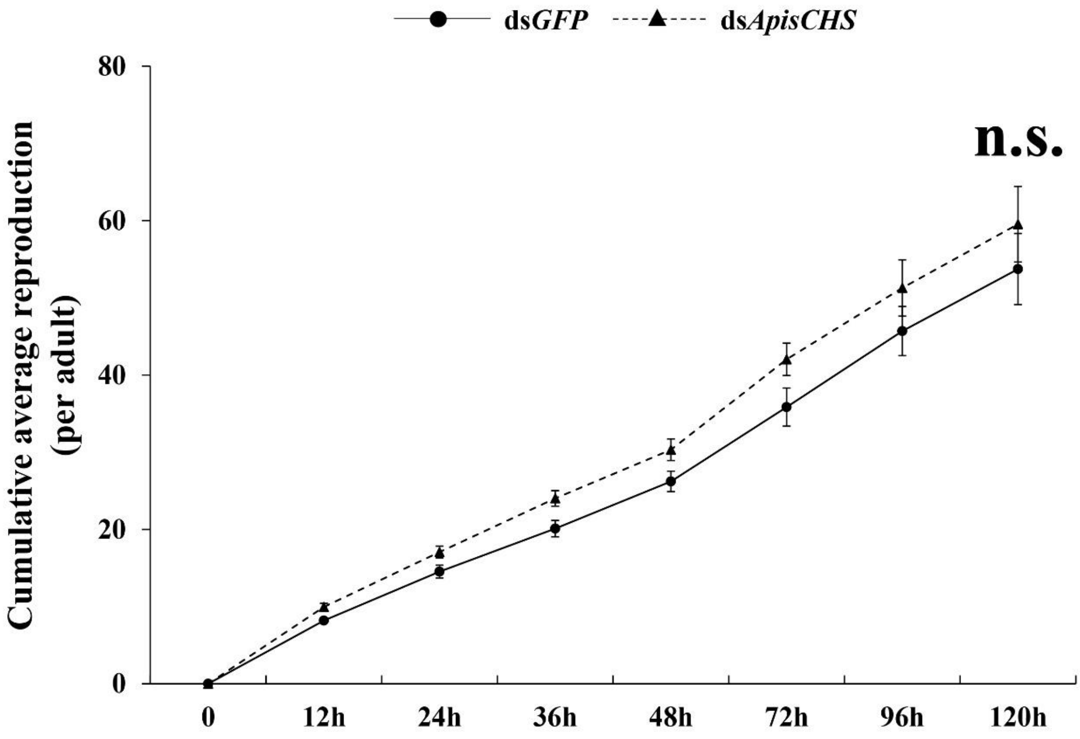

Figure S3. Cumulative average production after RNAi for adults

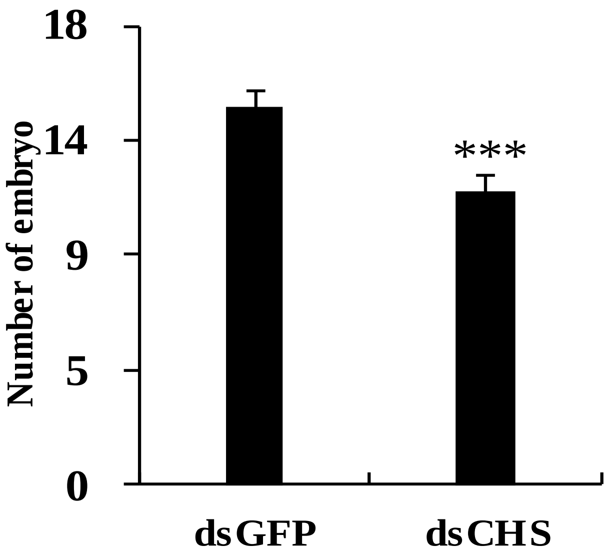

Figure S4. Numbers of embryos in treatment and control groups

A

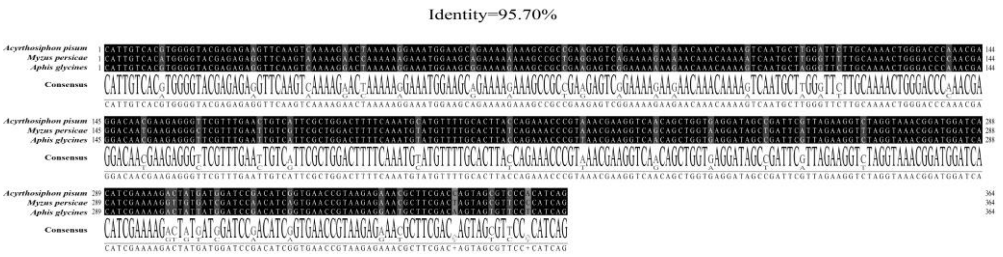

B

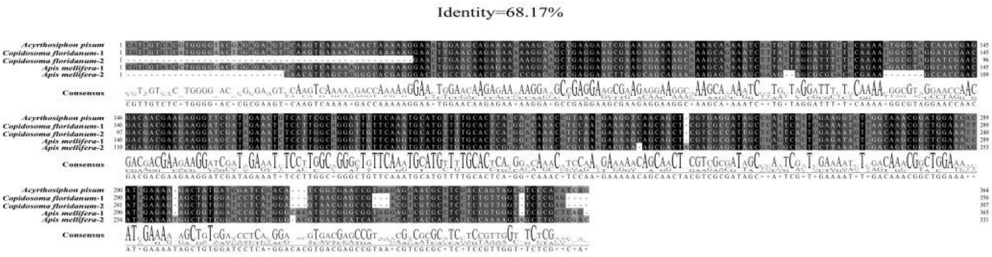

Figure S5. The similarity of these targeted fragments in aphids and beneficial insects

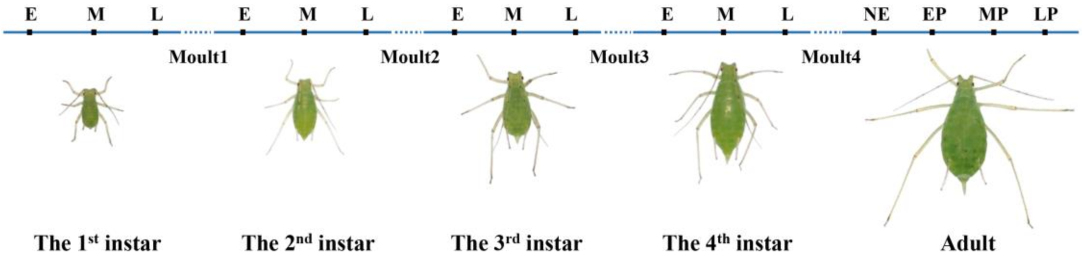

Figure S6. The sample collection at each developmental-point

A

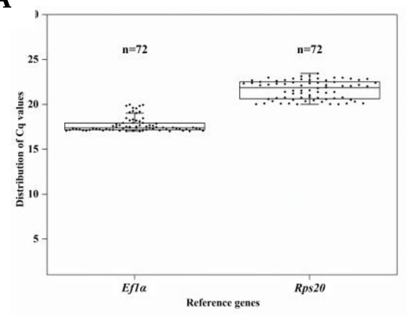

B

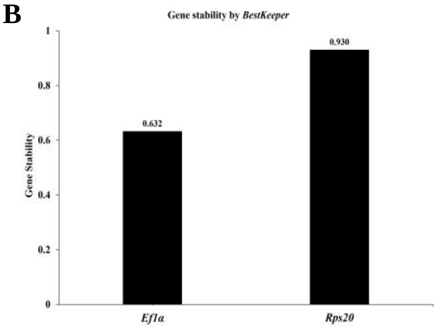

Figure S7. The stability of the reference genes among all the tested samples
